# Spike Triggered Covariance in Strongly Correlated Gaussian Stimuli

**DOI:** 10.1371/journal.pcbi.1003206

**Published:** 2013-09-05

**Authors:** Johnatan Aljadeff, Ronen Segev, Michael J. Berry, Tatyana O. Sharpee

**Affiliations:** 1 Computational Neurobiology Laboratory, The Salk Institute for Biological Studies, La Jolla, California, United States of America; 2 Center for Theoretical Biological Physics and Department of Physics, University of California, San Diego, La Jolla, California, United States of America; 3 Department of Life Sciences and The Zlotowski Center for Neuroscience, Ben-Gurion University of the Negev, Beer-Sheva, Israel; 4 Princeton Neuroscience Institute, Princeton University, Princeton, New Jersey, United States of America; Indiana University, United States of America

## Abstract

Many biological systems perform computations on inputs that have very large dimensionality. Determining the relevant input combinations for a particular computation is often key to understanding its function. A common way to find the relevant input dimensions is to examine the difference in variance between the input distribution and the distribution of inputs associated with certain outputs. In systems neuroscience, the corresponding method is known as spike-triggered covariance (STC). This method has been highly successful in characterizing relevant input dimensions for neurons in a variety of sensory systems. So far, most studies used the STC method with weakly correlated Gaussian inputs. However, it is also important to use this method with inputs that have long range correlations typical of the natural sensory environment. In such cases, the stimulus covariance matrix has one (or more) outstanding eigenvalues that cannot be easily equalized because of sampling variability. Such outstanding modes interfere with analyses of statistical significance of candidate input dimensions that modulate neuronal outputs. In many cases, these modes obscure the significant dimensions. We show that the sensitivity of the STC method in the regime of strongly correlated inputs can be improved by an order of magnitude or more. This can be done by evaluating the significance of dimensions in the subspace orthogonal to the outstanding mode(s). Analyzing the responses of retinal ganglion cells probed with 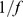 Gaussian noise, we find that taking into account outstanding modes is crucial for recovering relevant input dimensions for these neurons.

## Author Summary

In many areas of computational biology, including the analyses of genetic mutations, protein stability and neural coding, as well as in economics, one of the most basic and important steps of data analysis is to find the relevant input dimensions for a particular task. In neural coding problems, the spike-triggered covariance (STC) method identifies relevant input dimensions by comparing the variance of the input distribution along different dimensions to the variance of inputs that elicited a neural response. While in theory the method can be applied to Gaussian stimuli with or without correlations, it has so far been used in studies with only weakly correlated stimuli. Here we show that to use STC with strongly correlated, -type inputs, one has to take into account that the covariance matrix of random samples from this distribution has a complex structure, with one or more outstanding modes. We use simulations on model neurons as well as an analysis of the responses of retinal neurons to demonstrate that taking the presence of these outstanding modes into account improves the sensitivity of the STC method by more than an order of magnitude.

## Introduction

How do neurons encode sensory stimuli? One of the primary difficulties in answering this long-standing problem is the fact that sensory stimuli have high dimensionality. For example, responses of many visual neurons are affected by image patterns that require at least a 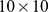 pixel grid for their description as well as a temporal history spanning multiple time bins or basis functions. Determining what input combinations affect the neural responses is a key step in characterizing the neural computation. Broadly speaking, to detect the presence of certain features in the environment over a range of distances and light conditions, one needs to disambiguate the presence of this feature at a weak contrast from the presence of a similar, but different feature presented at a higher contrast. This can only be achieved with nonlinear functions that depend on multiple input components, such as the presence of an edge of correct orientation and the absence of the edge orthogonal to it [1]. In support of these arguments, the responses of neurons in different sensory modalities are found to be sensitive to multiple input combinations. Examples include vision [2]–[7], audition [8]–[10], olfaction [11], somatosensation [12] and mechanosensation [13]. Neurons respond with all-or-none responses termed spikes. The goal of different methods for characterizing neural feature selectivity is to determine how the probability of eliciting a spike from a neuron depends on its inputs. The underlying assumption is that this dependence of spike probability on input parameters will have a low-dimensional structure. Finding either the linear input dimensions that modulate the spike probability (we will refer to these dimensions as relevant) or quadratic forms of inputs [14]–[16] is the focus of much of the current research in the field.

Much of the analysis of neural selectivity for multiple input combinations has been carried out using uncorrelated (“white noise”) or weakly correlated inputs. With such inputs, the relevant input dimensions can be found using a computationally inexpensive method known as spike-triggered covariance (STC) [6], [7], [17]–[22]. The STC method works by comparing the change in variance along different dimensions in the input space across all stimuli and across stimuli that elicited a spike. The dimensions along which the variance is found to be significantly different represent the relevant input dimensions for the response of a particular neuron. The method is not limited to strictly Gaussian inputs provided that the inputs are still circularly symmetric [23], which is another example of an input distribution without correlations.

In principle the STC method can also be used with correlated Gaussian stimuli [7], [20]. The case of correlated stimuli - especially with strong correlations, where the second moment of the covariance spectrum may be infinite - is important for neural coding. This is because signals in the sensory environment possess such correlations in both the second and higher orders [24]–[30]. Because the properties of a cell's relevant subspace may change depending on the stimulus statistics as a result of adaptation [31], [32], it may not be sufficient to study neural coding using uncorrelated stimuli. Here we show that with strongly correlated inputs, the significance analysis for determining which of the dimensions obtained by the STC method are relevant for neural spiking needs to be modified to take into account a rather complicated covariance structure of randomly selected inputs drawn from such input ensembles. The nonuniform covariance structure, which has properties akin to the graph laplacian in small-world networks [33], breaks the symmetry in the input space, and therefore may obscure many significant dimensions.

**Figure 1:**
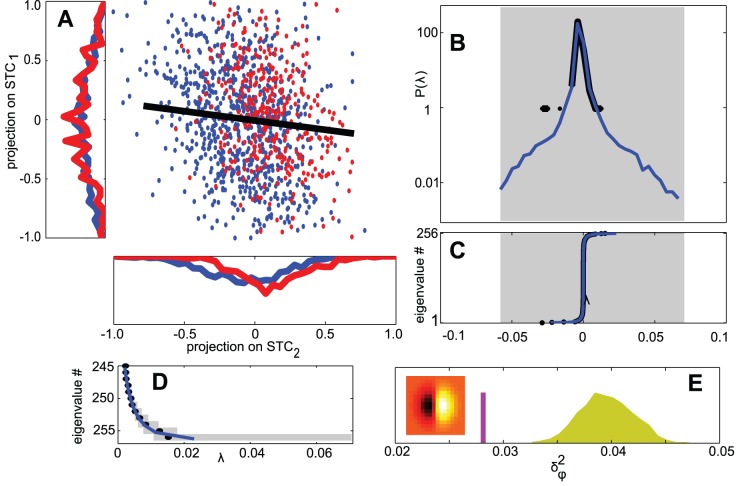
Spike triggered covariance analysis of simulated spike trains of a model with a single feature orthogonal to the coherent mode. (A) Inputs are plotted when projected on the eigenvectors corresponding to the largest eigenvalues of 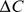 (in units of pixel illumination). Marginal distributions are plotted for each dimension. Inputs that elicited a spike are shown in red, and those that did not in blue. By construction, the change in variance is larger along the first dimension. (B) The empirical eigenvalue distribution of 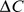 (black) compared to the null distribution (blue). No eigenvalues of 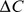 are found to be significant (shaded area indicates 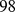
% confidence intervals for the support of the null distribution) (C) Rank ordered eigenvalues (black) plotted with the null distribution (blue). (D) Nested rank-wise significance testing. The highest ranked eigenvalues of 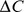 are within the 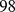
% confidence intervals derived from the null distribution constructed for each rank separately (see Materials and Methods for details). (E) For each random spike train we computed 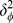, the variance of the projection of the spike-triggered stimulus on the relevant feature 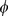 (distribution shown in yellow). The purple line indicates 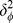 for the real spike train, suggesting the spike train contains enough signal to determine the relevant feature as significant. Inset shows the relevant feature, a 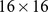 image patch (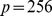). Simulation details: 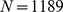, 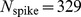, 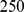 repetitions to find 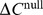.

The most prominent aspect of the natural scenes covariance structure is the presence of the so-called “coherent” mode [34]. This stimulus dimension approximately corresponds to the zero frequency input component and has a corresponding eigenvalue that is at least 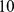 times larger than the mean eigenvalue of the input covariance matrix. Even in datasets of fairly large size, the extremely large variance along the coherent mode obscures many of the truly relevant dimensions for neural spiking (Fig. 1). These effects are also reproduced in our analysis of the responses of ganglion cells from the salamander retina probed with 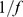-type naturalistic Gaussian stimuli. We identify a close relationship between the covariance structure derived from natural scenes to that defined by the Spiked-Wishart matrix model [35], [36]. This allows us to explain the effects in the context of the STC method using results from random matrix theory, and suggest ways to bypass sampling variability along the outstanding modes.

## Results

### Spike triggered covariance

Mathematically, the first step in the STC method is to compute the covariance matrix of stimuli that lead to a spike 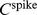 and the covariance matrix of all stimuli 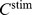:
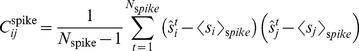
(1)

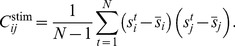
(2)Here, 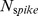 is the number of recorded spikes, 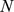 is the number of stimulus frames, 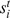 is the value of the stimulus along the 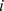
th dimension at time 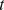, the *hat* denotes that this stimulus triggered a spike, the *bar* denotes the average across the input distribution and 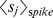 is the average across the distribution of inputs that triggered a spike (the so called “spike-triggered-average”).

As the second step, one computes the difference between these covariance matrices:
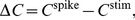
(3)and finds the eigenvalues that are significantly different from zero. The corresponding eigenvectors span the neuron's relevant subspace.

To determine statistical significance of the eigenvalues, they need to be compared to the null distribution, which is the distribution of eigenvalues of the matrices 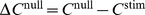. The matrices 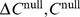 are formed assuming no association between the stimulus and the neural response, i.e. by using random spike times chosen at the same rate found for real neurons. If the spike train has particular temporal structure (e.g. bursting, a refractory period), the 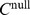 is obtained by random shifts of the spike train with periodic boundary conditions [20]. Significant eigenvalues of 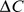 can be positive or negative. The procedures for determining statistical significance are detailed in Materials and Methods.

The final step of the STC method is to remove stimulus correlations from the estimate of dimensions found to be significant. This can be done by multiplying them with the (pseudo)inverse of 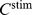 (see Materials and Methods). The method which we use to find the optimal rank of the pseudoinverse matrix is detailed in [22], [37] and for completeness described in Materials and Methods.

We note that this approach, Eqs. (1)–(3), of finding the relevant stimulus dimensions by diagonalizing 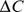 is equivalent to seeking eigenvectors of the following matrix [20]:
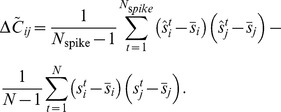
(4)This matrix describes a change in the second moment between the distributions stimuli that elicit a spike and that of all stimuli, after subtracting the mean stimulus 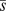. Despite the fact that 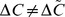, their eigenvectors coincide.

In another formulation, instead of subtracting the matrix 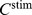 in Eq. (3), the stimulus is decorrelated (“whitened”) prior to its spike triggered characterization [7]. For completeness, the details of this method are brought in Materials and Methods. Throughout the manuscript, we will refer to this method as the “one centered” method, because the null distribution is centered around the identity matrix, rather than a matrix of zeros, as in Eq. (3). Correspondingly, we will refer to the version of the STC method obtained by diagonalizing Eq. (3) as the “zero-centered” method. In essence, both the one-centered and the zero-centered versions are similarly affected by inhomogeneous sampling variability.

The authors of [7] proposed a slightly different definition of the null distribution and a nested hypothesis technique for significance testing. For the model cell simulations we used both significance analysis methods, in both the “zero-centered” and “one-centered” STC formulations, and obtained similar results. For the rest of this paper we will refer to our significance testing method as the “global” one, and focus mainly on the “zero-centered” formulation of the STC method. Using this combination the important effects of the strong stimulus correlations on the analysis are more easily understood.

### Model cells presented with strongly correlated noise

We begin with an illustration of the problems that arise when the STC method is used to analyze neural responses to strongly correlated Gaussian noise (Fig. 1). We simulated a model neuron where the neuronal responses were modulated by stimulus projections onto a single dimension (termed here the relevant feature). The stimuli were constructed to match the second-order statistics from the set of images in the van Hateren dataset [38] (see Materials and Methods). In this example obtained for dataset of a moderate size, no eigenvalues fell outside of the 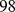
% confidence intervals (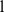
% significant bounds for the largest and smallest rank-ordered eigenvalues). Yet, the spike train contains enough signal about the cell's input-output function to identify the relevant feature for this level of significance. Specifically, the variance along the relevant dimension in the spike-triggered stimulus (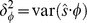) is much smaller than can be explained by random spike times (Fig. 1E).

### Outstanding modes in covariance matrices derived from natural stimuli

**Figure 2:**
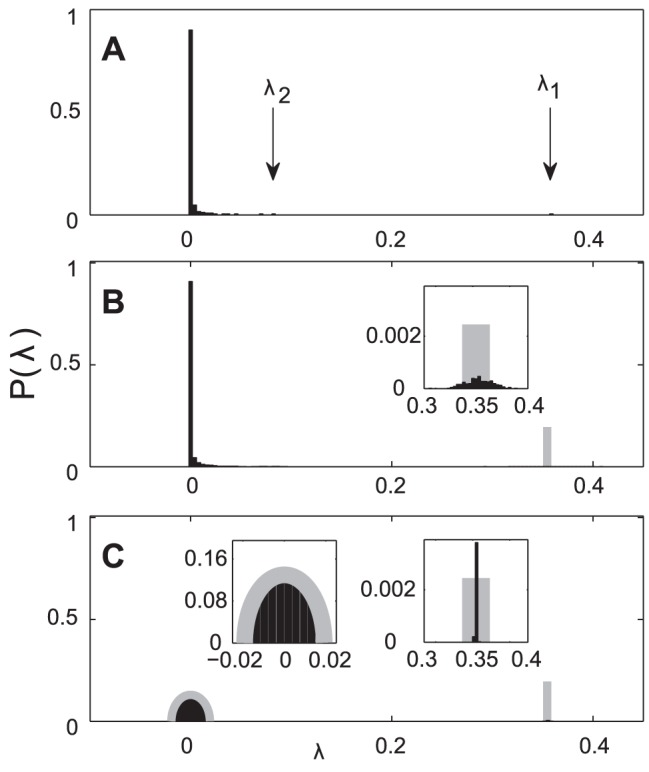
Spectra of the population covariance, sample covariance, and symmetric random matrices with matched element distribution. (A) Eigenvalue distribution of an example population covariance matrix 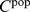 (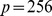) computed from the van Hateren data set. The largest eigenvalue (marked with an arrow) corresponds to an eigenvector with only positive components, and is 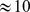 times larger than the second largest eigenvalue (also marked) and 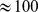 times larger than the mean eigenvalue. (B) Eigenvalue distribution of a collection of sample covariance matrices computed from stimuli randomly drawn from a multivariate Gaussian distribution 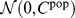. In gray is the analytic prediction for the outstanding eigenvalue. The spread of these eigenvalues (black, inset) is in agreement with the prediction in Eq. (8). (C) Eigenvalue distribution of symmetric random matrices with elements randomly drawn from a distribution given by the elements in the sample covariance matrices. In gray is the complete prediction (semicircle and outstanding eigenvalue) given by Eq. (5). Diagonal and off-diagonal elements are drawn separately from the distribution of matrix elements in panel B.

To understand the origin of such masking of the relevant feature(s), we consider the eigenstructure of covariance matrices for stimulus ensembles with strong pairwise correlations. For example, in the case of natural scenes that exhibit long range correlations over a very wide range of spatial scales [27], [39], principal component analysis (PCA) yields one outstanding eigenvalue (for example, see eigenvalue marked 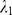 in Fig. 2A). The corresponding eigenvector has all positive components [28], [30] and is often referred to as the “coherent mode” [34]. To understand why such a coherent mode appears, one can consider the case where the correlations decrease only slightly over the range of image patches used to compute the covariance matrix. In this case, the correlation values in different image patches will be approximately the same. Such a matrix will have one outstanding eigenvalue with a corresponding eigenvector that has equal weights for all stimulus dimensions [40]. Small differences in the amount of covariation for pixel pairs with different spatial separation will lead to deviations in components of the coherent mode from each other, but the basic structure will remain the same as long as the mean of the correlation values exceeds the standard deviation of their fluctuations [40]. In fact, shuffling entries in the sample covariance matrices of natural stimuli yields matrices whose spectra follow the analytical predictions exactly [40], [41]. These analytical predictions generalize the Wigner semicircle law [42] for matrices whose elements have a non-zero mean:
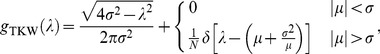
(5)where 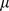 and 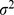 are the mean and variance of matrix elements. The distribution 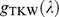 follows the semicircle law with the addition of one outstanding mode that appears once the mean of matrix elements exceeds their standard deviation. The eigenvector corresponding to the outstanding eigenvalue is 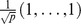. The semicircle law appears because matrices are no longer positive-definite after shuffling. However, the outstanding eigenvalue is located at exactly the same value as the outstanding eigenvalue of the natural scenes covariance matrix 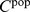 (see Fig. 2C).

In our analysis of the van Hateren database, the largest eigenvalue tends to be at least 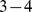 times larger than the second largest eigenvalue. This shows how strong the coherent mode is compared to other modes. The principal components ranked below the coherent mode form a collection of orthogonal “edge detectors”, some of which correspond to an eigenvalue still much larger than the mean eigenvalue of 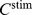, a signature of the stimulus' heavy-tailed covariance spectrum. Such large disparities in variance along the different dimensions in the stimulus space make it problematic to directly compare changes in variance induced by the observation of spikes along these different dimensions.

The detailed structure of sampling variability in the estimation of eigenvectors and eigenvalues can be understood in terms of the Spiked Wishart ensemble [35], [36]. In the Spiked Wishart matrix model, the true (population) covariance eigenvalues are all equal to one, except for a small number 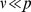 of outstanding modes with eigenvalues larger than one 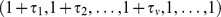, where 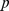 is the stimulus dimensionality. The distribution of sample covariance eigenvalues 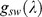 for a finite number of inputs has a positive bias, with the following analytical expressions [36]:
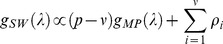
(6)

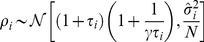
(7)

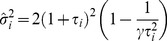
(8)where 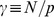 and 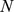 is the number of samples. The distribution 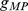 representing the “bulk” of eigenvalues is the so called Marčenko-Pastur distribution given by:
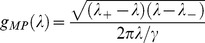
(9)

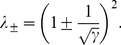
(10)This distribution corresponds to the sample covariance eigenvalues obtained when the true covariance is the identity matrix. Using numerical simulations we verified that, although the Spiked Wishart ensemble is only an approximation to the covariance matrices derived from natural stimuli, Eqs. (7) and (8) accurately describe the scaling of the variance and the mean of sample eigenvalues as 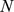 increases.

In addition to biases in eigenvalue estimates, there are also biases in the estimation of eigenvectors. The dot product between the true (population) 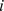
th eigenvector 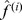 and the 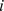
th eigenvector 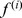 of the sample covariance approaches
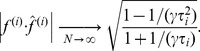
(11)


In other words, the “mixing” of the outstanding sample eigenvectors seen in Eq. (11) (note the dependence of this mixing on 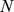 through 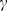) as well as the variance and bias in the sample eigenvalues seen in Eq. (7) means that whitening cannot be exact.

**Figure 3:**
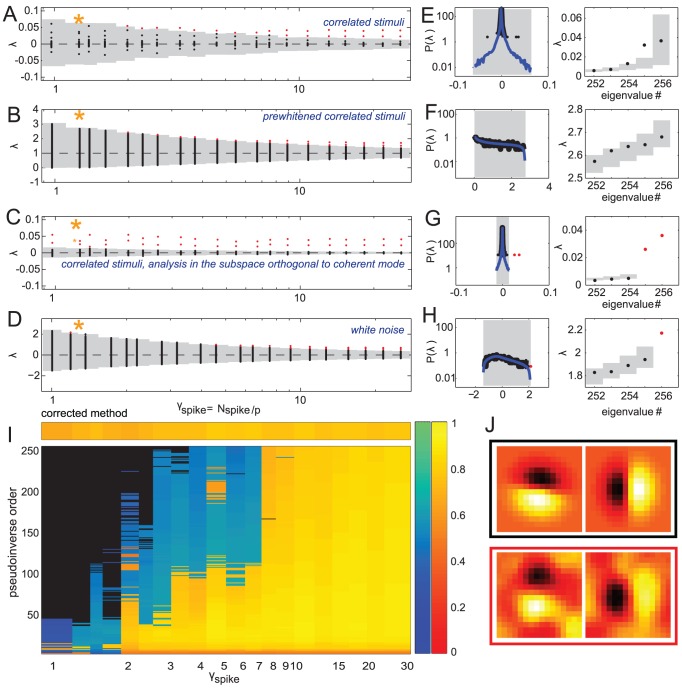
Spike triggered covariance analysis of a model neuron with two relevant features orthogonal to the coherent mode. Spectra of 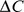 for increasing dataset size in the case of strongly correlated Gaussian noise (A–C) and white noise (D). (A–D) The range of spikes covered is from 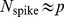 to 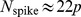. Panel B shows the results using the pre-whitening (“one-centered”) method, and panel C shows the results after evaluating significance in the subspace orthogonal to the coherent mode. Each vertical line shows the result of a single simulation. Significant (insignificant) eigenvalues are shown in red (black), and the range of the null distribution (1000 evaluations of 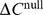, 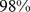 confidence interval) is shown in gray. (E–H, left) Gray shaded area is the support of the null distribution, which itself is plotted in blue. The significant (insignificant) portion of the spectrum of 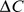 is plotted in red (black). These example spectra, with the corresponding significant vectors, are for conditions with a small number of spikes (indicated by an orange star in A–D) for which both of the formulations find no significant dimensions. 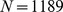, 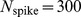 for the correlated stimulus condition in panels E–G, 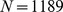, 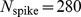 for the white stimulus condition in panel H. (E–H, right) Results of the nested significance testing. We note that the second ranked eigenvalue in panel E is outside of its confidence interval, but still cannot be found to be significant. This happens because of the noise along the coherent mode. (I) STC analysis using all pseudoinverse orders using the nested significance testing with 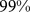 confidence intervals (large box) compared to the analysis using our proposed correction scheme. Black means no significant features were found for that combination of 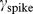 and pseudoinverse order. Cold (hot) colors indicate that one (two) features were found to be significant. The corresponding color bars on the right indicate the geometric average of the feature projections on the two model dimensions (cold colors) or the subspace overlap with the model, cf. Eq. (36) (hot colors). (J) Results when STC is performed using the proposed correction scheme. The two relevant dimensions (black frame) and the decorrelated significant features (red frame) have subspace overlap of 0.82. The models were defined such that the mean firing rate remained unchanged between the two stimulus conditions.

In the context of the spike triggered covariance the consequences of such properties of the distribution of sample eigenvalues are twofold. First, Eq. (8) indicates that the variance of the outstanding eigenvalues around their mean increases with the square of their value and is inversely proportional to the number of samples. Thus, for sample sizes that are not much larger than the stimulus dimensionality (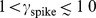 in the simulation results presented in Fig. 3A), the increased variance of the outstanding sample eigenvalue means that 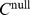 and 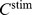 will not cancel each other exactly along that vector. Second, the mean estimate contains a positive bias relative to the population values, cf. Eq. (7). The combination of these two effects widens the null-distribution used to test the significance of the resulting eigenvectors, effectively masking features that should otherwise be identified as being relevant.

### Pre-whitening

One way to compensate for the symmetry breaking effects caused by strong correlations in the input space is to equalize variances before applying the STC method. This is the essence of the “one-centered” formulation of the STC method [7]. In principle, this “whitening” should work with Gaussian stimuli with any covariance structure. However, as discussed above, in the case of strongly correlated stimuli, the estimation of eigenvalues (i.e variances along different dimensions in the input space) possesses strong variability, cf. Eq. (7). As a consequence, normalization by a variance estimated from one part of the dataset does not fully remove correlations in a different subset of the data. With increasing dataset size, the estimate of the variance along the coherent mode improves. However, because the absolute value of variance is not relevant in the pre-whitening method, dimensions with smaller variance can cause just as much contamination as the coherent mode. In addition, the estimation of variance along dimensions corresponding to 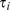 just larger than 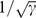 remains poor for large 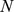. If 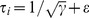 the sample eigenvalue estimation error diverges as 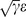, as follows from Eq. (8). In other words, as the number of samples 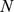 and 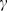 increase, the bulk of the distribution narrows, and new eigenvalues separate from the bulk. It is these eigenvalues with intermediate values that are poorly determined and make it problematic to equalize variance along different dimensions. Another signature of this phenomenon is that 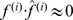 for these dimensions, as follows from Eq. (11). Thus, these dimensions are poorly estimated from the sample covariance and, as a consequence, the variance along one stimulus dimension in the training set will be inappropriately used to normalize variance along a different stimulus dimension in the test set. Altogether, we observed that pre-whitening stimuli did not improve the estimation of relevant stimulus features compared to the zero-centered method, compare panels A and B in Fig. 3. Intuitively, in the zero-centered method the dimensions with the largest variance provide the largest uncertainty in variance estimation, whereas in the one-centered version the problematic dimensions change depending on the dataset size, and are not easily identified *a priori*.

We have also explored the possibility of using a pseudoinverse of the covariance matrix instead of the full inverse to normalize variance along different dimensions (see Materials and Methods for details). When using the pseudoinverse (instead of the inverse), stimulus dimensions with small variance in the stimulus ensemble are removed to avoid noise amplification along these dimensions (see Materials and Methods for details). However, an immediate consequence of choosing a small pseudoinverse order 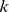 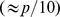 is that the stimulus dimensionality is reduced to 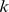. This implies that the effective 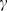 of the problem is now 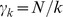, i.e. 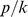 times larger than 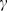. This could work well in some cases as illustrated in Fig. 3I. Here, in simulations based on a small number of spikes, the use of pseudoinverse can help recover one or two significant features while the standard zero-centered method fails to find any. However, the use of pseudoinverse only helps within a very narrow band of small pseudoinverse orders. This band may be difficult to determine when analyzing real neural data. In addition, this procedure limits the reconstruction to a linear combination of only a few leading stimulus dimensions. In many cases, the relevant features do include components along stimulus dimensions with smaller variance, and in those cases, the effective increase in 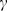 will not improve the performance of the STC method. Indeed, one observes that in cases where two significant dimensions are obtained by using substantial reduction in dimensionality of the pseudoinverse, the resulting dimensions have the subspace projection onto the model features of 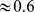 whereas this value is 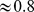 when using the full inverse and a larger number of spikes to obtain for a comparable effective 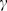 (Fig. 3I).

Finally, in the regime where 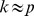 (i.e. “almost full” inverse), the prewhitening approach works just as well as the “zero-centered” formulation, and a relatively high value of the signal-to-noise ratio parameter 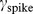 is required for recovery of the full relevant subspace.

### Correction scheme

As another way to compensate for the symmetry breaking effects caused by strong correlations in the input space, we propose to modify the “zero-centered” formulation of the STC method in the following way. Because the largest drop in variance is between the coherent mode and other dimensions, we propose here to test the significance of changes in variance separately along the coherent mode and in the subspace orthogonal to it. Explicitly, to do the analysis in the 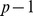 dimensional subspace, the coherent mode 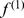 is projected out of all stimuli. If 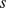 is a stimulus vector and 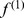 normalized to length 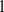, one can perform the STC analysis using 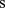 instead of 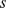 where:
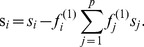
(12)


In this approach the correct number of relevant dimensions is determined by evaluating significance in the subspace orthogonal to the coherent mode and then adding back their projections on the coherent mode from the corresponding eigenvectors evaluated in the full input space (see below).

We find that considering the coherent mode separately from the rest of stimulus dimensions reduces the value of 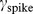 for which the full relevant subspace is found to be significant by a factor of 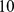 (Fig. 3C). This improvement can be approximated from Eqs. (7) and (8). Assuming the cell's relevant subspace is exactly orthogonal to the coherent mode, the extremal values of the null distribution are distributed as 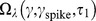. The variance of 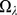 is:
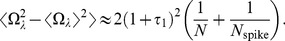
(13)This implies that the number of stimuli 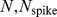 sufficient for identifying the relevant features as significant increases with 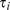 as:
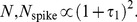
(14)Upon removal of the coherent mode, the minimum value of 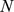 for which the signal to noise ratio will be high enough to identify the relevant dimensions scales as 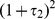 corresponding to the stimulus' second principal component. Therefore the improvement is proportional to 
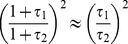
. In our simulations (Fig. 3A,C) this corresponds to a predicted 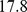 fold improvement. Given that our model features were not exactly orthogonal to the coherent mode, and that the spectrum obtained from the van Hateren dataset has a heavy tail and does not conform exactly to the Spiked Wishart ensemble, an approximate 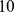 fold improvement represents a good agreement with the prediction.

It is noteworthy that the minimum requirement on the dataset size for obtaining the correct number of relevant dimensions is actually smaller with correlated stimuli than it is for white noise stimuli for the same neuron (compare Fig. 3 panels A–D) when the model parameters were matched such that the firing rate remains constant across different stimuli statistics. Another important point is that considering the coherent mode separately is different from simply discarding a “DC-like” component that could be found to be significant by the STC. This is because when 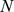 is small, no dimensions are found to be significant with the coherent mode as part of the stimulus ensemble (Fig. 1).

An important consideration is that the final analysis can include the components of the relevant dimensions onto the coherent mode. This is possible for two reasons. First, the coherent mode does not represent an arbitrary dimension in the input space but is one of the eigenvectors of the sample covariance matrix. Second, the significant eigenvectors of 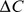 have a form 
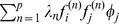
, where 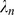 is the 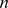
th eigenvalue corresponding to the 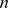
th eigenvector 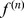 of the sample covariance matrix, and 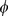 describes one of the relevant features [20]. Because of these two properties, eigenvectors evaluated in the full input space and in the subspace orthogonal to the coherent mode differ only in their components along the coherent mode (see Materials and Methods for the details of the derivation). This makes it possible to analyze cells with features that have nonzero components along the coherent mode.

**Figure 4:**
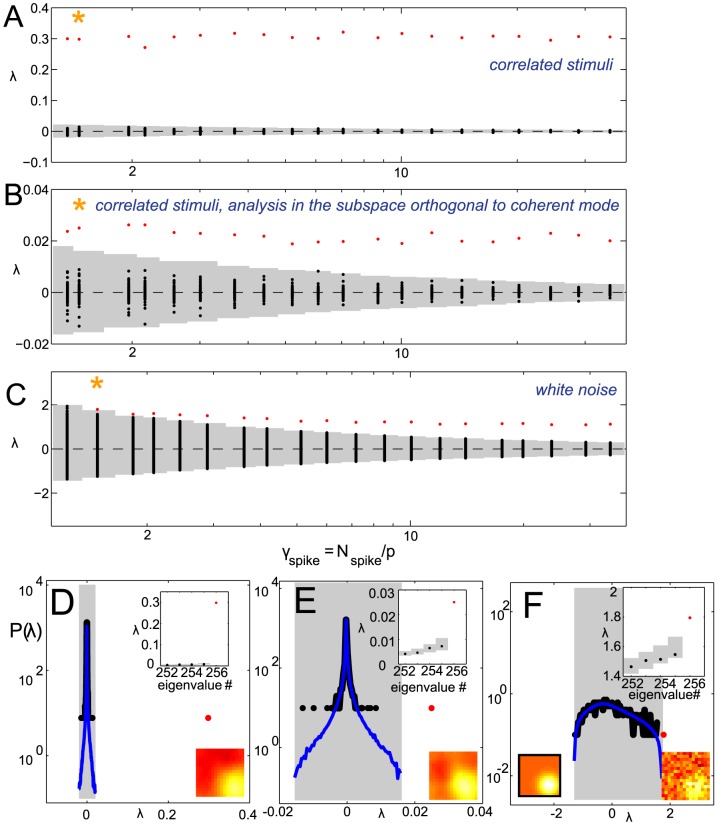
Spike triggered covariance analysis of a model neuron with one relevant feature that has a large component along the coherent mode. Spectra of 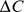 for increasing dataset size in the case of strongly correlated Gaussian noise (A,B) and white noise (C). (A–C) The range of spikes covered is from 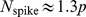 to 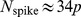. Panel B shows the results after evaluating significance in the subspace orthogonal to the coherent mode. Each vertical line shows the result of a single simulation. Significant (insignificant) eigenvalues are shown in red (black), and the 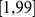 percentile range of the null distribution (1000 evaluations of 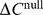) is shown in gray. (D–F) Gray shaded area is the support of the null distribution, which itself is plotted in blue. The correct feature is found using both stimulus conditions and both the original formulation of the STC method and our proposed correction. 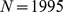, 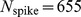 for the correlated stimulus condition in panels D and E, 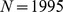, 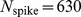 for the white stimulus condition in panel F (indicated by an orange star in panels A–C). Insets to panels D–F show the recovered feature (decorrelated in panels D,E) and the nested significance testing which does not affect the results. Black framed inset to panel D shows the model feature. The models were defined such that the mean firing rate remained unchanged between the two stimulus conditions.

We have verified that this approach also works in a large number of cases where the relevant stimulus dimensions have a large projection on the coherent mode (Fig. 4). One concern is that when such neurons are probed with a relatively small number of stimuli, then projecting the coherent mode out may “push” the relevant feature into the null eigenvalue distribution. This does not appear to be a problem in our simulations for 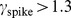 (Fig. 4B). If this does happen, the relevant subspace should be the one spanned by both the eigenvectors found to be significant in the full stimulus space and those found to be significant in the subspace orthogonal to the coherent mode.

### Application to retinal ganglion cells

**Figure 5:**
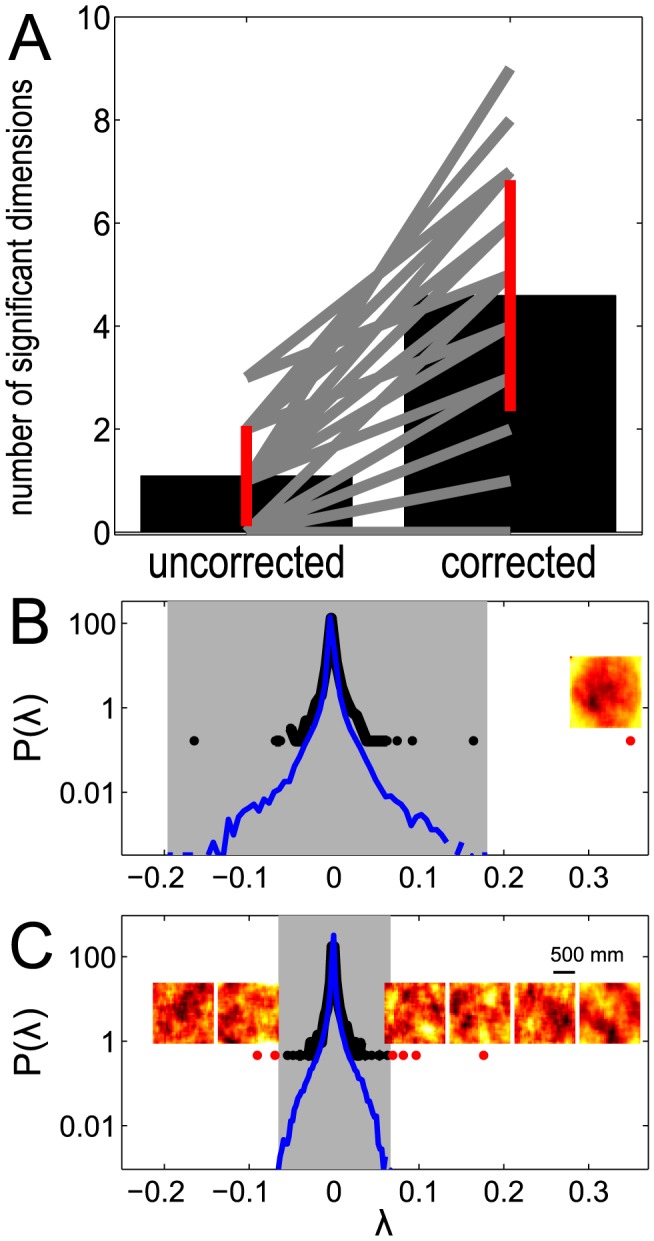
Analysis of salamander retinal ganglion cells. (A) Number of significant dimensions for 22 RGCs presented with a strongly correlated stimulus, before (*left*) and after (*right*) using our correction scheme. Gray lines represent single cells, and red lines represent the standard deviation. (B) Significant (insignificant) eigenvalues of 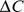 in red (black) and the null eigenvalue distribution (blue) for an example cell. Null distribution were constructed using the global approach with 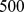 shuffled spike trains. Without the correction scheme there is only one significant dimension. The corresponding visual feature is shown in the inset before decorrelation. (C) Same for the corrected covariance matrix 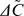. Here there are six significant dimensions with spots likely representing the subunits within the cell's receptive field. The gray shade indicates the range of the null distribution used to determine significance.

We now demonstrate the importance of this correction scheme by analyzing recordings of 22 salamander retinal ganglion cells (RGCs). These neurons were probed with a correlated noise stimulus whose covariance matrix was matched to that of natural visual stimuli. Without correcting for the presence of the coherent mode, the STC analysis yielded no significant dimensions for a third of the cells, and very few for the rest (Fig. 5). This happens because the eigenvalue corresponding to the coherent mode injects large eigenvalues into the null eigenvalue distribution (as seen in Eq. (8)), thus masking the cell's true relevant features. Following the correction, the number of significant dimensions per cell increased from 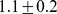 to 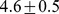 (see Fig. 5A for the full population values). The dimensionality of the relevant subspace increased for 21 out of 22 cells. For one cell, we were unable to find a significant dimension either before or after the correction of the method. The distributions of null eigenvalues used to determine which of the eigenvectors of 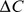 are significant (Fig. 5B,C) became much more narrow when evaluated in the subspace orthogonal to the coherent mode.

## Discussion

The goal of this work was to extend the range of applicability of a computationally simple method of spike-triggered covariance to strongly correlated stimuli. While the STC method in principle can be used with strongly correlated Gaussian stimuli, our results show that the inhomogeneous sampling variability can in practice make it difficult to recover the correct relevant subspace. We have characterized the effects generated by strong Gaussian correlations using simulations of two model neurons in a wide range of dataset sizes (which could also be viewed as an inverse measure of the neuron's level of internal noise). Results from random matrix theory, and specifically the Wigner and Spiked Wishart ensembles, suggest that the origin of these issues can be traced to the estimation bias and variance of covariance matrices with vastly different eigenvalues. We demonstrate that by considering the coherent mode, which corresponds to the largest eigenvalue, separately from the rest of stimulus dimensions, one can improve the method's sensitivity by 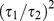.

One qualitative lesson offered by these analyses is that while the bulk of the eigenvalues of 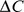 is a good proxy for the width of the null distribution in the case of white noise inputs, but not in the case of strongly correlated inputs.

Furthermore, our analysis suggests that sampling variability along the secondary outstanding modes corresponding to the next few principal components may have similar masking effects to the ones reported here for the coherent mode. Possible solutions to the full problem may include performing a sequence of analyses in subspaces of decreasing dimensionality, orthogonal to several leading principal components. However, the payoff from this procedure is (at most) of order 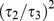 which in our case is 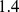. At the same time, one runs the risk of losing the ability to resolve the remaining dimensions because of the reduced signal to noise ratio.

Another potential solution is to correct for the estimation bias and variance in eigenvalues and eigenvectors, described by Eqs. (7) and (8). However, this procedure is difficult computationally and in most cases can only be done for simple eigenvalue distributions [43].

The treatment of the artifacts caused by a large coherent mode present in the data has been previously discussed in analyses of stock-markets [44], [45], evolution of proteins [46], and Human Immunodeficiency Virus (HIV) mutations [34]. In these cases, the extra dimension was removed and the resulting covariance structure was compared against the Marčenko-Pastur eigenvalue distribution that assumes no correlation between the variables and uniform variable variances. The case of reverse correlation experiments discussed here is different from these analyses because the spike triggered ensemble is compared to the full stimulus distribution. In addition, our analyses provide two important novel contributions. First, we show there is a crucial difference between discarding the coherent mode and projecting it out. This is because of the way the coherent mode injects noise into the null distribution. Second, the approach described here also permits the inclusion of the components of the relevant dimensions along the coherent mode in the final results. We hope that the ideas for treating the coherent mode presented here will also be relevant in other areas of computational biology.

## Materials and Methods

### Ethics statement

Experimental data were collected using procedures approved by the Institutional Animal Care and Use Committee of Princeton University, and in accordance with National Institutes of Health guidelines. Experimental and surgical procedures have been described previously [47].

### Stimulus

Each stimulus frame 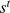 was randomly drawn from a multivariate Gaussian distribution with zero mean and covariance matrix 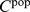, 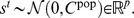 In the correlated stimulus case, the population covariance 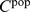 was computed from the covariance of 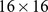 pixels patches from the van Hateren image database [38] (with no downsampling). In the uncorrelated (“white”) case, 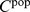 was the identity matrix.

### Testing for significance

We describe two approaches for determining significance of candidate features that were previously described in the literature: global and nested. When applied to our datasets, both of the approaches yielded similar results.

#### Global null distribution

Eigenvalues of 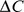 are identified as significant if they lie outside the 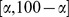 percentile interval of the null distribution, where parameter 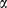 specifies the level of significance. The null distribution is constructed by computing many realizations of the matrix 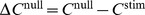.

If 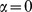 is used, the number of randomized spike trains is inversely proportional to the confidence interval with which significance is determined.

To describe how matrix 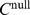 is computed, we recall that
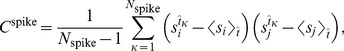
(15)where 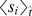 is the spike-triggered average of the 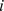
th stimulus component and spike times are denoted as 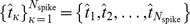.

The matrix 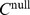 is computed in the exact same manner, but instead of the spike train 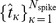, we use random spike trains 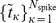:
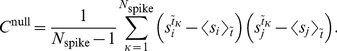
(16)Note that there are just as many random spike times 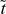 as real spike times 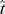. Moreover, when the spike train has a meaningful temporal structure, the random spike train can be obtained by a random shift of 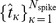, defined for all 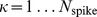 and for a random integer 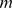 chosen uniformly between 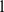 and 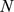 [20]:
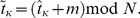
(17)Using all the realizations of 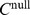 computed (i.e. all the randomly chosen 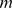
's), the eigenvalues of 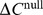 compose the null distribution.

#### Nested significance testing

Significance can also be tested in a nested fashion using 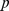 rank-ordered null distributions. This method is detailed in [7]. For completeness we briefly describe it here. For each of the randomized spike trains the eigenvalues of 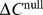 (or 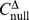 for the pre-whitened formulation, see Eq. (25) below) are rank ordered. The eigenvalues of each order from all the randomized spike train compose a separate null distribution. Then, for each of these 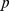 distributions we found a confidence interval. If the smallest eigenvalue of 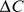 (or 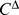) is smaller than the lower bound of its relevant confidence interval (or if the largest eigenvalue is larger than the upper bound) the corresponding eigenvectors are determined to be significant. These eigenvectors are then projected out of the stimulus and the analysis is repeated until no eigenvalues are found to be significant. Note that according to this method some eigenvalues of 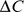 (or 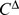) can identified as insignificant but still be outside of the confidence interval computed for their rank if the largest eigenvalue lies within its confidence intervals (see Fig. 3E, right).

### Decorrelation within the STC method

Within the STC method, stimulus correlations need to be removed from the estimates of eigenvectors obtained by diagonalizing matrix 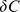. This correction is needed, because the eigenvectors of 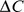 have a form 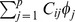, where 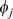 describe components of one of the relevant features [20]. As described above, one may wish to use a pseudoinverse, instead of the full inverse of the matrix 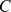 to minimize noise amplification at higher spatial frequencies. Assuming that the eigenvalues are ordered to be monotonically decreasing, the pseudoinverse of order 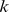 is given by
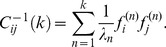
(18)


In the analysis of data from retinal ganglion cells, the optimal order of the pseudoinverse was determined in the following way. The dataset was divided into the training and test sets. The features were computed by diagonalizing the matrix 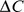, cf. Eq. (3), in either the full input space or in the space orthogonal to the coherent mode using the training set. Following that, the optimal pseudoinverse order 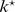 was selected as the one that yielded decorrelated features that convey the most information about, or give the largest predictive power for, the neural response. Explicitly,
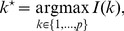
(19)

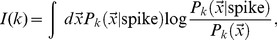
(20)where 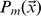 is the probability distribution of the projections of stimuli onto the 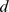 significant eigenvectors (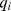), decorrelated by 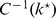. 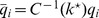 are the decorrelated significant features, and:
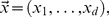
(21)

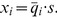
(22)


### Pre-whitening

As an alternative to removing stimulus correlations from the eigenvectors of 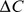, one can remove stimulus correlations from each of the stimulus vectors, prior to the diagonalization of 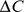, a procedure that is known as pre-whitening [7].

The sample stimulus covariance matrix from Eq. (2) can be written in terms of eigenvalues 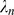 and eigenvectors 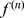 as
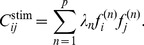
(23)We can now define a matrix 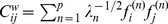. Then, the analogue of 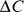 in the “one-centered” formulation is given by:
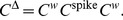
(24)This procedure is equivalent to whitening each of the stimulus frames independently (by multiplying it with 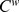) and then computing the spike-triggered covariance.

In the limit of infinite data, the null hypothesis corresponds to 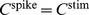. In this case 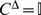. For a dataset of finite size, the null distribution is computed from many realizations of the matrix
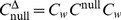
(25)where 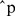 is defined by Eq. (16). The eigenvalues of 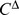 (most of which are close to 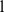) can then be compared to the null eigenvalue distribution, using either the nested or global comparison tests described above.

In Fig. 3 we analyzed the simulated spike trains using every pseudoinverse order of 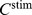. The prewhitening is then done using this matrix 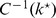 Eq. (18) instead of the full rank matrix 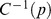.

Performing the pre-whitened STC analysis using all pseudoinverse orders is equivalent to testing 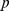 models. Therefore, the confidence interval of the null distribution should be adjusted from the 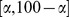 percentile range to 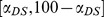, where 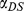 is the Dunn-Šidák correction:
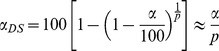
(26)


### Relevant features in the full stimulus space

We recall that according to Ref. [20], the significant eigenvectors of 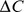 can be written as
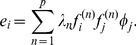
(27)


Thus, the eigenvectors of 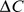 represent a sum of projection operators onto the principal components of the stimulus ensemble. When we perform the STC method in the subspace orthogonal to the first principal component of the stimulus, the eigenvectors of 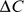 can be written as
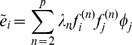
(28)(the coherent mode is exactly the vector 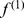).

Comparing expressions for the eigenvectors of 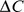 and 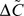, one observes that there is a one-to-one correspondence between them. This correspondence can be identified based on proportionality in components along second, third, and other principal components:
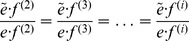
(29)for any 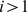. In sum, once the eigenvector 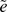 is found to be significant in the subspace orthogonal to 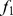, the eigenvector that should be identified as significant in the full stimulus space is 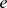 that satisfies the condition of Eq. (29).

### Model neurons

The nonlinearity was chosen to be a logistic function because such functions maximize the cell's noise entropy and thus minimize the assumptions imposed on the cell's response [48]. Using the models, we generated simulated spike trains in response to either a white or a correlated noise stimulus.

The model used in Fig. 3 (model “
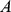
”) had a two dimensional relevant subspace with features orthogonal to the coherent mode. The probability of spiking was modeled to increase when the projection of the stimulus on either of the preferred features was large in absolute value (representing a logical OR function). If 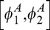 are the preferred model features and 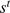 is the stimulus presented at time 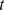 (here, the 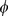
's and 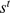 are 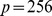 dimensional vectors) then the probability of a spike at time 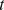 is:
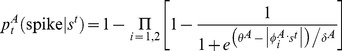
(30)where 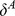 and 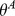 are parameters that determine the width and (soft) thresholds of the sigmoid nonlinearities for the model.

We have also considered the case where the projection of the stimulus on the features was not taken in absolute value, corresponding to a monotonic nonlinearity. In that case (model “
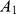
”, used in Fig. 1) the model was one dimensional, so the probability of a spike is
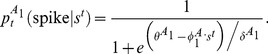
(31)


The effects described above were observed for both symmetric (Fig. 3) and monotonic (Fig. 1) nonlinearities.

The second model had one relevant input feature 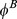 with a large component along the coherent mode. In this case, the probability of a spike was modeled as:
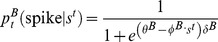
(32)where 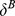 and 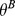 are the width and the threshold of the sigmoid nonlinearity of this model.

In units of the standard deviation of the projection of the stimulus on the model features (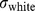, 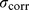) the model parameters were chosen to be:
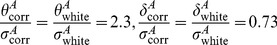
(33)

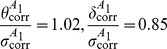
(34)

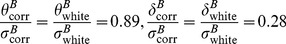
(35)


### Subspace overlap

The overlap measure we use when the dimensionality of the relevant subspace is greater than one is given by [49]:
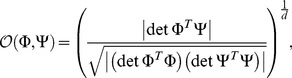
(36)where 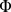 and 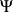 are 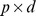 matrices that hold the model and computed features, respectively, 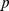 is the input dimensionality, and 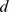 is the number of relevant features in the model.
